# Characterization of Lgr6+ Cells as an Enriched Population of Hair Cell Progenitors Compared to Lgr5+ Cells for Hair Cell Generation in the Neonatal Mouse Cochlea

**DOI:** 10.3389/fnmol.2018.00147

**Published:** 2018-05-14

**Authors:** Yanping Zhang, Luo Guo, Xiaoling Lu, Cheng Cheng, Shan Sun, Wen Li, Liping Zhao, Chuijin Lai, Shasha Zhang, Chenjie Yu, Mingliang Tang, Yan Chen, Renjie Chai, Huawei Li

**Affiliations:** ^1^ENT Institute and Otorhinolaryngology Department of Affiliated Eye and ENT Hospital, State Key Laboratory of Medical Neurobiology, Fudan University, Shanghai, China; ^2^Key Laboratory for Developmental Genes and Human Disease, Ministry of Education, Institute of Life Sciences, Southeast University, Nanjing, China; ^3^Department of Otolaryngology Head and Neck Surgery, Jiangsu Provincial Key Medical Discipline Laboratory, Affiliated Drum Tower Hospital, Nanjing University Medical School, Nanjing University, Nanjing, China; ^4^Jiangsu Province Hi-Tech Key Laboratory for Bio-Medical Research, Southeast University, Nanjing, China; ^5^Co-innovation Center of Neuroregeneration, Nantong University, Nantong, China; ^6^Key Laboratory of Hearing Medicine, National Health and Family Planning Commission (NHFPC), Shanghai, China; ^7^Shanghai Engineering Research Center of Cochlear Implant, Shanghai, China; ^8^The Institutes of Brain Science and the Collaborative Innovation Center for Brain Science, Fudan University, Shanghai, China; ^9^Institutes of Biomedical Sciences, Fudan University, Shanghai, China

**Keywords:** Wnt, Lgr5, Lgr6, proliferation, differentiation, hair cell, inner ear, RNA-Seq

## Abstract

Hair cell (HC) loss is irreversible because only very limited HC regeneration has been observed in the adult mammalian cochlea. Wnt/β-catenin signaling regulates prosensory cell proliferation and differentiation during cochlear development, and Wnt activation promotes the proliferation of Lgr5+ cochlear HC progenitors in newborn mice. Similar to *Lgr5*, *Lgr6* is also a Wnt downstream target gene. Lgr6 is reported to be present in adult stem cells in the skin, nail, tongue, lung, and mammary gland, and this protein is very important for adult stem cell maintenance in rapidly proliferating organs. Our previous studies showed that Lgr6+ cells are a subpopulation of Lgr5+ progenitor cells and that both Lgr6+ and Lgr5+ progenitors can generate Myosin7a+ HCs *in vitro*. Thus we hypothesized that Lgr6+ cells are an enriched population of cochlear progenitor cells. However, the detailed distinctions between the Lgr5+ and Lgr6+ progenitors are unclear. Here, we systematically compared the proliferation, HC differentiation, and detailed transcriptome expression profiles of these two progenitor populations. We found that the same number of isolated Lgr6+ progenitors generated significantly more Myosin7a+ HCs compared to Lgr5+ progenitors; however, Lgr5+ progenitors formed more epithelial colonies and more spheres than Lgr6+ progenitors *in vitro*. Using RNA-Seq, we compared the transcriptome differences between Lgr5+ and Lgr6+ progenitors and identified a list of significantly differential expressed genes that might regulate the proliferation and differentiation of these HC progenitors, including 4 cell cycle genes, 9 cell signaling pathway genes, and 54 transcription factors. In conclusion, we demonstrate that Lgr6+ progenitors are an enriched population of inner ear progenitors that generate more HCs compared to Lgr5+ progenitors in the newborn mouse cochlea, and the our research provides a series of genes that might regulate the proliferation of progenitors and HC generation.

## Introduction

Hearing loss is a permanent sensory disorder affecting all populations throughout the world, and sensory hair cell (HC) loss is the main reason for permanent hearing loss (Bohne et al., 1976; Kelley, 2006). In non-mammalian vertebrates, HCs are able to regenerate to recover lost hearing function through direct differentiation and mitotic regeneration (Corwin and Cotanche, 1988; Ryals and Rubel, 1988; Stone and Cotanche, 2007). In mammals, the vestibular system has the capacity for only very limited spontaneous HC regeneration (Forge et al., 1993; Warchol et al., 1993; Kawamoto et al., 2009; Burns et al., 2012; Golub et al., 2012), and the neonatal mouse cochlea still has some progenitors that have a limited ability to regenerate HCs if they are damaged during early neonatal development (Jan et al., 2013; Cox et al., 2014). However, upon maturation, HC loss in mammals tends to be irreversible because HC progenitors gradually lose their regenerative capacity as the animal ages and because of the complex organization of progenitors and other cells within the fully developed organ of Corti (Oesterle et al., 2008; Warchol, 2011).

During cochlear development, both HCs and supporting cells (SCs) originate from prosensory progenitor cells that express the cyclin dependent kinase inhibitor p27 kip1 (Cdkn1b) (Chen and Segil, 1999). Prosensory regions can be identified at embryonic day (E)11. The progenitors of HCs and SCs are still proliferating at E12 (Ruben, 1967), but between E13 and E14 most of the cells in the prosensory region begin to exit the cell cycle (Ruben, 1967). The progenitors located in the apical cochlear duct exit the cell cycle earlier than those in the basal cochlear duct (Ruben, 1967). Starting at E13.5, the progenitors in the mid-basal cochlear duct begin to differentiate into HCs and to spread bidirectionally (Chen et al., 2002); the formation of HCs is finished when the entire length of the cochlear epithelium is patterned into three outer rows of HCs and one inner row between E17 and E18 (Chen and Segil, 1999; Chen et al., 2002). HC maturation is finished when the key proteins, such as prestin, are expressed in the outer HCs (Belyantseva et al., 2000). It was reported that isolated SCs in the neonatal cochlea have the ability to proliferate and differentiate into HCs *in vitro* (White et al., 2006; Sinkkonen et al., 2011), although the postnatal sensory HCs and SCs are postmitotic *in vivo*. SCs in the mouse cochlea and utricle have been regarded as a reliable source of HC regeneration after damage (Li et al., 2003, 2015; White et al., 2006; Lin et al., 2011; Sinkkonen et al., 2011; Golub et al., 2012; Cox et al., 2014; Wang et al., 2015). Wnt/β-catenin signaling plays important roles by regulating the differentiation and proliferation of prosensory cells in cochlear development (Chai et al., 2012; Shi et al., 2012), and when Wnt/β-catenin signaling is knocked out or inhibited sensory progenitor cells cannot proliferate and differentiate into HCs (Jacques et al., 2012; Shi et al., 2014). *Lgr5*, one of the Wnt downstream target genes, is expressed in a subset of SCs in the cochlea (Chai et al., 2011), and Lgr5+ cells have been identified as an enriched HC progenitor population compared to the rest of the SC population in many studies (Chai et al., 2012; Shi et al., 2012).

Similar to *Lgr5*, *Lgr6* is also a Wnt downstream target gene, and it is present in adult stem cells in the skin, nail, tongue, lung, and mammary gland (Snippert et al., 2010; Oeztuerk-Winder et al., 2012; Ren et al., 2014; Lehoczky and Tabin, 2015; Blaas et al., 2016). Lgr6 is very important for adult stem cell maintenance in rapidly proliferating organs. In the adult mouse skin, Lgr6+ cells can proliferate and differentiate into all skin cell lineages, and they function in wound repair (Snippert et al., 2010). Lgr6+ cells give rise to the nails during homeostatic growth, and they play a role during digit tip regeneration (Lehoczky and Tabin, 2015). In the tongue, Lgr6+ stem cells can generate mature taste cells within the taste papillae *in vitro* (Ren et al., 2014). In the lung, E-Cad/Lgr6+ cells can self-renew and differentiate into bronchial and alveolar tissue (Oeztuerk-Winder et al., 2012). In the mammary gland, adult Lgr6+ stem cells can sustain alveologenesis throughout multiple pregnancies (Blaas et al., 2016).

In our previous study, we found that Lgr6 was only expressed in the inner pillar cells (IPs) from the embryonic to the neonatal period in the mouse cochlea and that these cells were a distinct subpopulation of Lgr5+ progenitors (Zhang et al., 2015). When we isolated the Lgr6+ cells by flow cytometry, they could generate Myosin7a+ HCs *in vitro*, and thus we hypothesized that Lgr6+ cells are an enriched cochlear progenitor population that generate HCs more efficiently than Lgr5+ progenitors. However, the detailed proliferation and differentiation ability of Lgr6+ progenitors has not been studied yet, and the differences between the Lgr5+ and Lgr6+ progenitors has been unclear.

In this study, we undertook a comprehensive comparison between the Lgr5+ and Lgr6+ progenitors. We found that Lgr6+ progenitors had a significantly greater capacity to generate Myosin7a+ HCs than Lgr5+ progenitors, while Lgr5+ progenitors had a greater capacity to proliferate than Lgr6+ progenitors. RNA-seq was performed to explore the differences of transcriptome expression profiles between these two HC progenitors, and we identified a set of significantly differentially expressed genes that might play roles in the proliferation and differentiation of HC progenitors. In summary, our data demonstrate that Lgr6+ progenitors are an enriched population of HC progenitor cells, and we provide a set of candidate genes that might serve as new potential therapeutic targets for HC generation.

## Materials and Methods

### Experimental Animals

Transgenic mice were purchased from Jackson Laboratory, including Lgr5-EGFP-IRES-creERT2 (Stock #008875) and Lgr6-EGFP-IRES-CreERT2 mice (Stock #016934). DirectPCR Lysis Reagent (Mouse Tail) (102-T, Viagen) was used to isolate genomic DNA from mouse tail tips. Genotyping of transgenic mice was performed as in our previous study (Zhang et al., 2015). All protocols were approved by the Animal Care and Use Committee of Fudan University and were consistent with the National Institutes of Health Guide for the Care and Use of Laboratory Animals. We made all efforts to minimize the number of animals used and alleviate their suffering.

### Isolation of Lgr5+ Progenitors and Lgr6+ Progenitors via Flow Cytometry

The isolation of HC progenitors was performed as in previous studies (Zhang et al., 2015; Waqas et al., 2016; Cheng et al., 2017). Briefly, postnatal day (P)0–P3 mouse sensory epithelium samples were digested by 0.125% trypsin/EDTA (Invitrogen) in 0.01M PBS for 9 min at 37°C. The reaction was terminated by soybean trypsin inhibitor (10 mg/ml; Worthington Biochem). Following mechanical trituration with about 80–100 strokes with blunt tips (Eppendorf, #22491245), isolated cells were percolated through a 40 μm cell strainer (BD Biosciences). Non-viable cells were labeled by propidium iodide (1 μg/ml, Sigma). Dissociated cells were then sorted using the GFP channel on a MoFlo^®^ SX FACS cytometer (Beckman Coulter), and the EGFP+ cells were collected. Isolated cells were lysed in RLT buffer (Qiagen) for quantitative real-time PCR (q-PCR) assay or stored at -80°C until RNA-Seq analysis. For each RNA-Seq library, nearly 10,000 sorted cells were used. Re-sort analysis, immunofluorescence, and q-PCR were used to confirm the identity of the sorted cells.

### Sphere-Forming Assay and Differentiation Assay

The sphere-forming assay and differentiation assay were performed as in previous studies (Waqas et al., 2016; Cheng et al., 2017). For the sphere-forming assay, sorted Lgr5+ and Lgr6+ cells were cultured separately at a density of 2 cells/μl in low attachment dishes (Costar, 3599) in DMEM/F12 medium supplemented with N2 (1%, Invitrogen, 17502), B27 (2%, Invitrogen, 17504), EGF (20 ng/ml, Sigma, E9644), IGF (50 ng/ml, Sigma, I8779), heparan sulfate (20 ng/ml, Sigma, H4777), β-FGF (10 ng/ml, Sigma, F0291), and ampicillin (0.1%, Sigma, A9518). After 5 days in culture, the numbers of spheres were quantified. Then the spheres were plated on laminin/polylysine (1:1)-coated 4-well dishes and cultured for 10 days in DMEM/F12 media with N2, B27, and 0.1% ampicillin for the differentiation assay. For the direct differentiation assay, flow cytometry-isolated Lgr5+ and Lgr6+ cells (at a density of 20 cells/μl) were plated on laminin/polylysine coated dishes and cultured for 10 days in DMEM/F12 media with N2, B27, and 0.1% ampicillin. EdU (10 ng/ml, Sigma, F0291) was added in the medium to measure the proliferation of the Lgr5+ and Lgr6+ cells. After 10 days, the cells and spheres were fixed and immunostained for different markers.

### Cryosection, Immunostaining, and Image Acquisition

Cryosections were made according to a previous study (Zhang et al., 2015). In brief, P3 cochleae from Lgr6-EGFP-Ires-CreERT2 and Lgr5-EGFP-Ires-CreERT2 mice were fixed in 4% paraformaldehyde (PFA), and a Leica CM3050S microtome was used to make the cryosections. The spheres and isolated cells were fixed overnight at 4°C in 4% PFA. Non-specific binding was blocked with 10% donkey serum in 0.01 M PBS for 1 h at 37°C. Samples were incubated overnight at 4°C with primary antibodies, including anti-Myo7a (Proteus Bioscience, #25-6790, 1:1,000 dilution), anti-Sox2 (Santa Cruz, #sc-17320, 1:500 dilution), anti-EGFP (Abcam, 1:1,000 dilution), and anti-cytokeratin (Sigma-Aldrich, C2562, 1:200 dilution). After rinsing with 0.01 M PBS three times, the samples were incubated for 1 h at 37°C with corresponding secondary antibodies, including antibodies conjugated with FITC, Cy3, or Cy5 (1:500 dilution, Jackson ImmunoResearch). 4,6-diamidino-2-phenylindole (DAPI, 1:800 dilution, Sigma-Aldrich) was used to label cell nuclei. Cell proliferation was labeled with the Click-it EdU imaging kit (Invitrogen). Images were captured with a Leica SP8 confocal microscope. ImageJ (NIH) and Photoshop CS4 (Adobe System) were used to analyze the images.

### Quantitative Real-Time PCR

The total RNA from the flow cytometry-isolated GFP+ cells was extracted with an RNeasy micro kit (Cat. #74004, Qiagen). A PrimerScript II 1st Strand cDNA Synthesis Kit (Cat. #6210A, TaKaRa) was used to synthesize the cDNA. q-PCR was performed using SYBR Premix Ex Taq II (Tli RNaseH Plus) (Cat. #RR820A, TaKaRa) on an AB 7500 Real-Time PCR System. The ΔΔCT method was used to analyze the gene expression level. Primers for the q-PCR assay are listed in **Supplementary Table S1**.

### RNA-Seq and Data Analysis

Approximately 10,000 FACS-isolated Lgr5+ or Lgr6+ cells from P3 cochleae were dissolved in RNALater. The extracted RNA from Lgr5+ or Lgr6+ cells was split into three fractions for separate replicates. The SMART-Seq v4 Ultra Low Input RNA Kit and Illumina mRNA-Seq Sample Prep Kit were used for library preparation, and library quality was analyzed using an Agilent Bioanalyzer. An Illumina HiSeq2500 Platform was used to generate 150-bp paired-end sequences. Fastq format RNA-Seq reads were trimmed using Trimmomatic. We mapped the clean reads to the mouse reference genome (mm9) using TopHat (Trapnell and Schatz, 2009). Transcripts and genes were annotated according to the RefGene database (NCBI). Differential gene expression analysis was performed using Cufflinks. Genes with *p*-values < 0.05 were noted as significantly differentially expressed.

### Statistical Analyses

GraphPad Prism software was used for statistical analyses. Two-tailed, unpaired Student’s *t*-tests were used to determine statistical significance. Data are shown as the means ± SE, and *p* < 0.05 was considered statistically significant.

## Results

### Lgr6 Was Expressed in a Subpopulation of Lgr5+ Progenitors in P3 Cochleae

**FIGURE 1:**
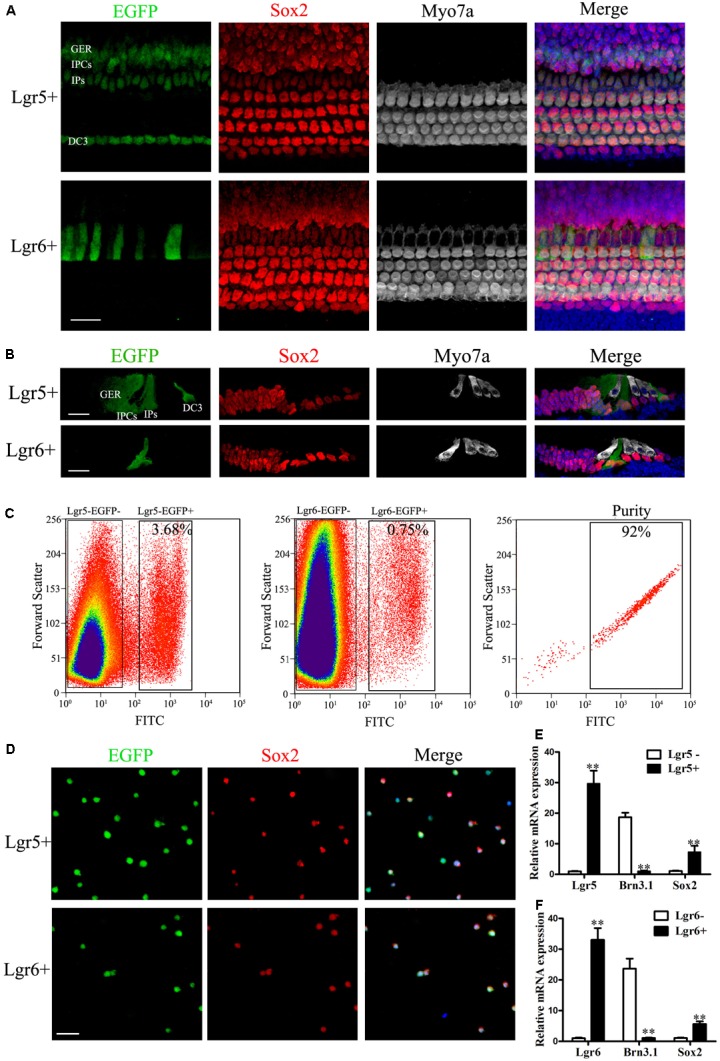
Re-sort analysis, immunostaining, and q-PCR of flow-sorted Lgr5+ and Lgr6+ cells from the postnatal cochlea. **(A)** At P3, Lgr5 was expressed in the third row of Deiters’ cells (DC3), the inner pillar cells (IPs), the inner phalangeal cells (IPCs), and the lateral GER, while Lgr6 was only expressed in the IPs. **(B)** Cryosection showed that Lgr5 was expressed in DC3s, IPs, IPCs and the GER, and Lgr6 was only expressed in a subset of IPs in the P3 organ of Corti. **(C)** GFP+ cells and GFP– cells were isolated using flow cytometry. Re-sort analysis of GFP+ cells demonstrated > 90% purity. **(D)** Immunostaining of Lgr5+ cells and Lgr6+ cells from the cochlea showed a high percentage of Sox2+ (95.4% and 95.2%, respectively) and GFP+ (95.8% and 96.6%, respectively) cells, and no Myo7a+ cells, among the sorted cells. **(E,F)** q-PCR showed that isolated Lgr5+ cells and Lgr6+ cells had significantly higher Lgr5 and Lgr6 expression, slightly higher Sox2 expression, and significantly lower Brn3.1 expression compared to the Lgr5- cells and Lgr6– cells, respectively. Scale bars are 20 μm. ^∗∗^*p* < 0.01.

First, we assessed the expression pattern of Lgr5 and Lgr6 in the P3 mouse sensory epithelium using Lgr5-EGFP-Ires-CreERT2 and Lgr6-EGFP-Ires-CreERT2 mice. Consistent with our previous studies (Chai et al., 2011; Zhang et al., 2015), immunohistochemical results showed that Lgr5 was expressed in the IPs, the inner phalangeal cells (IPCs), the third row of Deiters’ cells (DC3), and the lateral greater epithelial ridge (GER) in the whole mounts and cryosections of the sensory epithelium (**Figures 1A,B**), and the Lgr5 expression pattern was similar from the apex to the base in the cochlear duct (**Supplementary Figure S2**). Lgr6 was only expressed in a subset of the IPs, which are a subpopulation of Lgr5+ progenitors (**Figures 1A,B**), and Lgr6 was only expressed in the basal and middle turns of the organ of Corti (**Supplementary Figure S2**).

Previous studies showed that both Lgr5+ and Lgr6+ progenitors can generate HCs *in vitro* (Chai et al., 2012; Zhang et al., 2015). In order to determine the HC generation ability and proliferation ability of these two progenitor populations, we isolated the Lgr5+ and Lgr6+ progenitors via flow cytometry from P0–P3 Lgr5-EGFP-Ires-CreERT2 and Lgr6-EGFP-Ires-CreERT2 transgenic mice. We found that around 3.68% of the isolated cells were Lgr5+ and around 0.75% of the isolated cells were Lgr6+ (**Figure 1C**). Immunofluorescent staining showed that 95.8% of the Lgr5+ progenitors (**Figure 1D**) and 95.6% of the Lgr6+ progenitors (**Figure 1D**) sorted by flow cytometry were GFP+. Isolated Lgr5+ and Lgr6+ progenitors showed no staining for the HC marker Myo7a (data not shown), but nearly all cells showed staining for the SC marker Sox2 (95.4 and 95.2%, respectively) (**Figure 1D**). q-PCR was performed immediately after FACS to determine the purity of FACS-isolated Lgr5+ and Lgr6+ cells, and the results showed that the Lgr5+ and Lgr6+ progenitors had significantly higher Lgr5 and Lgr6 expression, respectively, and both populations had higher Sox2 expression and lower HC marker Brn3.1 expression compared to Lgr5- and Lgr6- cells (*p* < 0.05, **Figures 1E,F**). These results confirmed the high purity of the sorted Lgr5+ and Lgr6+ progenitors.

### Lgr6+ Progenitors Generate Significantly More HCs Than Lgr5+ Progenitors *in Vitro*

**FIGURE 2:**
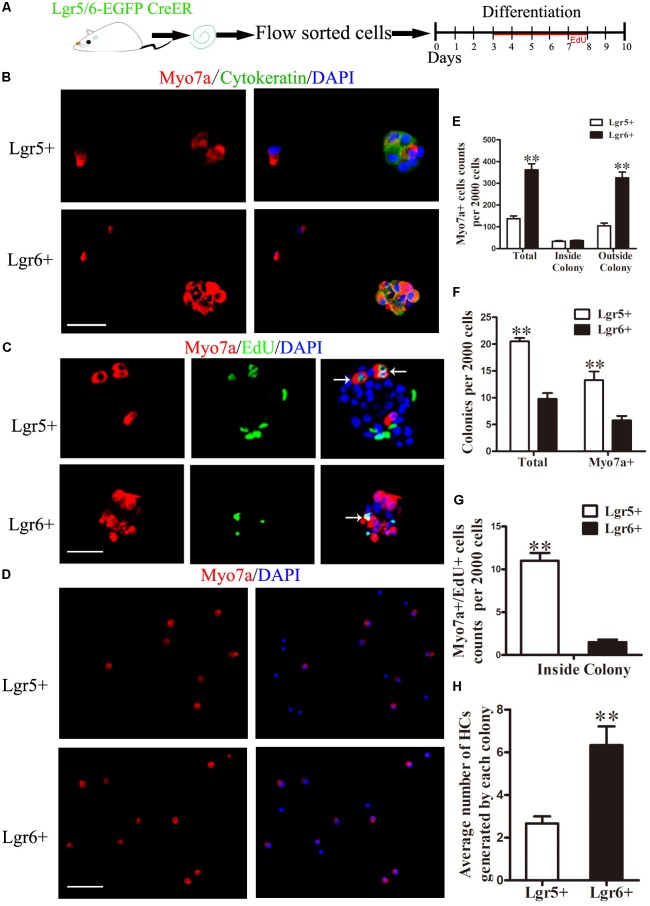
Lgr6+ progenitors generate more HCs compared to Lgr5+ progenitors *in vitro.* **(A)** Schematic depicting the experimental process. We dissected the cochleae and then cultured the sorted GFP+ cells at 20 cells/μl for 10 days. **(B)** Both Lgr5+ and Lgr6+ progenitors could generate Myo7a+ colonies. **(C)** Both Lgr5+ and Lgr6+ progenitors could generate Myo7a+/EdU+ cells inside of the colonies. **(D)** Both Lgr5+ and Lgr6+ progenitors could generate Myo7a+ cells outside the colonies. **(E)** The total number of Myo7a+ cells in each well per 2,000 cells and the numbers of Myo7a+ cells inside and outside the colonies. The Lgr5+ progenitors formed around 140 Myo7a+ cells per 2,000 cells and about 33 Myo7a+ cells inside the colonies, while the Lgr6+ progenitors formed around 360 Myo7a+ cells per 2,000 cells and about 36 Myo7a+ cells inside the colonies. **(F)** The numbers of colonies in each well per 2,000 cells. The Lgr5+ progenitors formed around 20 colonies and 10 Myo7a+ colonies, while the Lgr6+ progenitors formed around 13 colonies and 6 Myo7a+ colonies. **(G)** Lgr5+ progenitors generated significantly more Myo7a+/EdU+ cells than Lgr5+ progenitors inside of the colonies (11.0 ± 0.91 and 1.5 ± 0.29 HCs, respectively). **(H)** Each Myo7a+ colony from the Lgr6+ progenitors generated significantly more HCs than the Myo7a+ colonies from Lgr5+ progenitors. ^∗∗^*p* < 0.01, *n* = 4. Scale bars are 20 μm.

The differentiation assay used in this study was a standard method to investigate the differentiation and proliferation ability of HC progenitors as previously reported (Chai et al., 2012; Shi et al., 2012; Waqas et al., 2016; Cheng et al., 2017). To explore the HC generation capability of Lgr5+ and Lgr6+ progenitors, 2,000 sorted GFP+ cells were cultured in polylysine-laminin coated 4-well dishes at a density of 20 cells/μl in serum-free medium for 10 days. EdU was added to label the mitotically generated cells from day 3 to day 8, and the cells were then immunostained with antibodies against the HC marker Myo7a (**Figure 2A**). The results showed that both Lgr5+ and Lgr6+ progenitors could generate Myo7a+ cells and Myo7a+ colonies (*n* = 4, **Figures 2B–D**), and we found that the same number of Lgr6+ progenitors generated significantly more HCs than Lgr5+ progenitors (361.8 ± 28.35 HCs and 137.5 ± 12.7 HCs, respectively) (*p* < 0.01, *n* = 4, **Figures 2D,E**). We also used the epithelium marker cytokeratin to label the colonies and found that compared with the Lgr5+ progenitors, the Lgr6+ progenitors generated more HCs outside of the colonies, which represent the directly differentiated HCs (104 ± 13.72 HCs and 325.5 ± 29.85 HCs, respectively, per 2,000 cells) (*p* < 0.01, *n* = 4, **Figure 2E**). However, when we counted the cytokeratin-positive colonies, we found that the Lgr5+ progenitors generated significantly more colonies than the Lgr6+ progenitors (20.5 ± 0.65 colonies and 9.75 ± 1.11 colonies, respectively, per 2,000 cells) (*p* < 0.01, *n* = 4, **Figure 2F**). Moreover, the number of Myo7a+ colonies generated from the Lgr5+ progenitors was also significantly greater than the number of Myo7a+ colonies generated from the Lgr6+ progenitors (13.25 ± 1.65 Myo7a+ colonies and 5.75 ± 0.85 Myo7a+ colonies, respectively, per 2,000 cells) (*p* < 0.01, *n* = 4, **Figure 2F**). However, each Myo7a+ colony from the Lgr6+ progenitors generated significantly more HCs than the Myo7a+ colonies from Lgr5+ progenitors (6.30 ± 0.68 HCs and 2.5 ± 0.58 HCs, respectively, per colony) (*p* < 0.01, *n* = 4, **Figure 2H**). When we counted the Myo7a and EdU double positive (Myo7a+/EdU+) cells, we observed Myo7a+/EdU+ cells inside of the colonies in both Lgr5+ and Lgr6+ progenitors. Meanwhile, Lgr5+ progenitors generated significantly more Myo7a+/EdU+ HCs than the Lgr6+ progenitors (11.0 ± 0.91 HCs and 1.5 ± 0.29 HCs, respectively, per 2,000 cells) (*n* = 4, **Figure 2G**). Thus, together the total number of Myo7a+ HCs inside of the colony, which represents the mitotically generated HCs, was not statistically different between the Lgr5+ and Lgr6+ progenitors (33.0 ± 3.67 HCs and 36.25 ± 2.18 HCs, respectively, per 2,000 cells) (*n* = 4, **Figure 2E**). When we calculated the percentages, we found that the majority of the HCs generated from Lgr6+ progenitors were outside of the colony (89.78%), while the HCs generated from Lgr5+ progenitors were 75.91% outside of the colony and 24.09% inside of the colony. Together, these results demonstrated that Lgr6+ progenitors generated significantly more HCs than Lgr5+ progenitors and that Lgr6+ progenitors generated HCs mainly through direct differentiation, while Lgr5+ progenitors generated HCs through both direct differentiation and mitotic generation. This also indicates that the Lgr5+ populations other than inner pillar cells are responsible for the mitotic HC generation.

### Lgr5+ Progenitors Have Greater Sphere-Forming Ability, While Lgr6+ Progenitors Have Greater HC Generation Ability *in Vitro*

**FIGURE 3:**
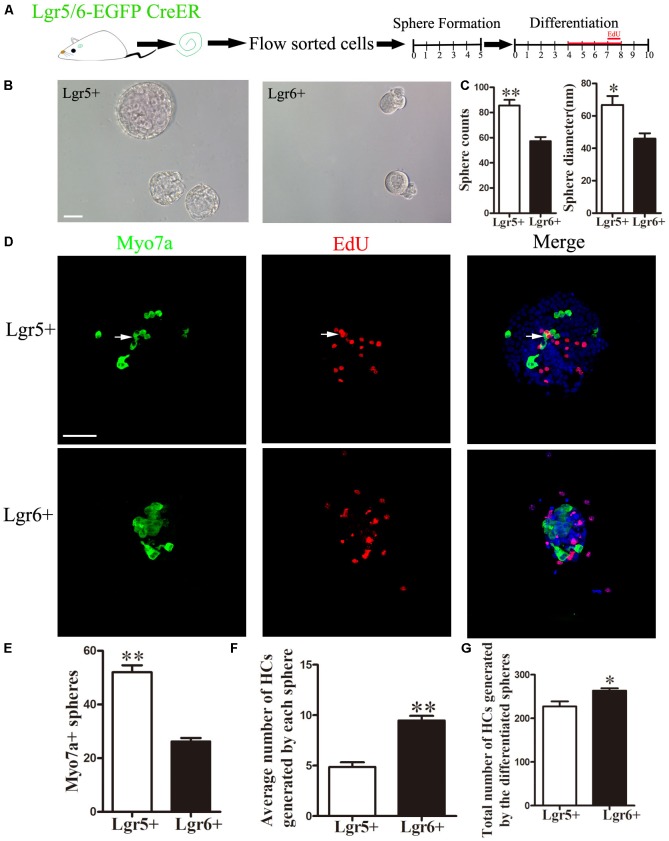
Lgr5+ progenitors have greater sphere-forming ability than Lgr6+ progenitors, and Lgr6+ progenitors have greater differentiation ability than Lgr5+ progenitors **(A)** Schematic depicting the experimental procedure. Cochleae were dissected, and flow-sorted Lgr5+ and Lgr6+ progenitors were cultured for 5 days in ultra low-attachment dishes, after which the spheres were transferred to laminin/polylysine (1:1)-coated 4-well dishes and cultured for another 10 days with EdU added to the medium from day 4 to day 8. **(B)** Both Lgr5+ and Lgr6+ progenitors could generate spheres. **(C)** Lgr5+ progenitors generated significantly more spheres than Lgr6+ progenitors, and spheres from Lgr5+ progenitors had a significantly larger diameter than those from the Lgr6+ progenitors. **(D)** Differentiated spheres from Lgr5+ progenitors generated Myo7a+ HCs and Myo7a+/EdU+ double-positive cells (arrows). Differentiated spheres from Lgr6+ progenitors also generated Myo7a+ HCs. **(E)** Lgr5+ progenitors generated significantly more Myo7a+ spheres than those from Lgr6+ progenitors. **(F)** Each differentiated sphere from Lgr6+ progenitors generated significantly more Myo7a+ HCs than those from Lgr5+ progenitors. **(G)** The 2,000 differentiated spheres from Lgr6+ progenitors generated significantly more Myo7a+ HCs than those from Lgr5+ progenitors. ^∗^*p* < 0.05; ^∗∗^*p <* 0.01, *n* = 4. Scale bars are 50 μm.

It has been reported that postnatal Lgr5+ progenitors can proliferate and form spheres when cultured in serum-free medium (Chai et al., 2012; Shi et al., 2012). In order to specifically compare the proliferative ability between Lgr5+ and Lgr6+ progenitors, we performed a sphere-forming assay. A total of 2,000 isolated Lgr5+ and Lgr6+ progenitors (2 cells/μl) were cultured in 24-well ultra low-attachment plates for 5 days in the medium that is suitable for sphere forming (**Figure 3A**). The number and the diameter of spheres generated from Lgr5+ or Lgr6+ progenitors were measured to determine the proliferation capacity of HC progenitors (**Figure 3B**). Both the numbers and the diameters of the spheres generated from Lgr5+ progenitors were significantly greater than spheres generated from the Lgr6+ progenitors (*p* < 0.05, *n* = 4, **Figure 3C**). To further evaluate the HC generation capacity of the spheres derived from Lgr5+ and Lgr6+ progenitors, we collected the spheres and allowed them to differentiate in serum-free medium for another 10 days (**Figure 3A**). Then we quantified the number of Myo7a+ HCs in all differentiated spheres generating from the 2,000 isolated cells and in each differentiated sphere (**Figures 3F,G**). Among the spheres generated from Lgr5+ progenitors, around 60.47% contained Myo7a+ HCs, while 45.61% of the spheres generated from Lgr6+ progenitors contained Myo7a+ HCs (**Figures 3D,E**). Moreover, in the spheres containing Myo7a+ HCs, our results showed that each Lgr6+ sphere generated significantly more Myo7a+ HCs compared to the Lgr5+ spheres (9.77 ± 0.45 HCs/sphere and 4.38 ± 0.48 HCs/sphere, respectively) (*p* < 0.01, *n* = 4, **Figure 3F**). In total, the 2,000 Lgr6+ progenitor cells generated significantly more Myo7a+ HCs than the same number of Lgr5+ progenitors (263.7 ± 5.78 HCs and 227.3 ± 11.36 HCs, respectively) (*p* < 0.05, *n* = 4, **Figure 3G**). These results indicated that Lgr5+ progenitors have greater proliferation and sphere-forming capacity, while Lgr6+ progenitors have greater HC generation ability *in vitro*.

### Analysis of RNA-Seq Results

In order to shed light on the mechanism involved in the proliferation and differentiation of Lgr5+ and Lgr6+ progenitors, we collected 10,000 FACS-isolated Lgr5+ and Lgr6+ cells from P3 cochleae to perform gene expression analysis using RNA-Seq to investigate differentially expressed genes between Lgr5+ and Lgr6+ progenitors. Between 11.9 million and 28.0 million paired-end reads were obtained for each sample, in which 51.0–75.7% of the reads were mapped correctly to the reference genome (mouse mm9). Every gene expression was measured by FPKM (Fragments Per Kilobase of transcript per Million fragments mapped) as in a previous report (Chen et al., 2017). The top 200 most abundant genes in the Lgr5+ and Lgr6+ progenitors are shown in **Supplementary Figure S1**.

### Differentially Expressed Genes in Lgr5+ and Lgr6+ Progenitors

**FIGURE 4:**
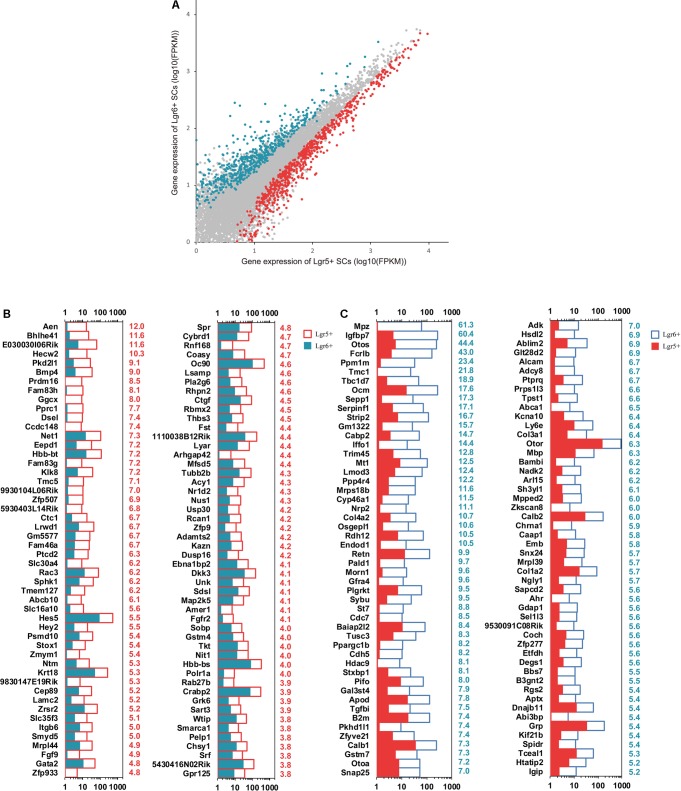
Differentially expressed genes in Lgr5+ progenitors and Lgr6+ progenitors. **(A)** X-Y scatter plot showing differentially expressed genes in Lgr5+ progenitors and Lgr6+ progenitors. The red dots represent highly expressed genes in Lgr5+ progenitors, and the blue dots represent highly expressed genes in Lgr6+ progenitors. **(B)** The 100 most differentially expressed genes in Lgr5+ progenitors. The numerical values in red on the right side of each panel represent the fold difference in expression in Lgr5+ progenitors versus Lgr6+ progenitors. **(C)** The 100 most differentially expressed genes in Lgr6+ progenitors. The numerical values in blue on the right side of each panel represent the fold difference in expression in Lgr6+ progenitors versus Lgr5+ progenitors.

Top differentially expressed genes in these two HC progenitors were selected for further analysis. The expression levels above background and at least 1.5-fold different between these two groups were defined as differentially expressed genes (**Figure 4A**, *p* < 0.05). We found that there were 1,157 and 862 differentially highly expressed genes in Lgr5+ progenitors and Lgr6+ progenitors, respectively. The top 100 differentially expressed genes between these two progenitors are shown in **Figures 4B,C**. The functions of some genes have been studied in the sensory epithelium, including *Hes5*, *Hey2*, *Gata2*, *Fgfr2*, and *Stox1*, which were highly expressed in the Lgr5+ progenitors, and *Adk* and *Otoa*, which were highly expressed in the Lgr6+ progenitors. However, the expression of most differentially expressed genes and their functions in the ear have not been reported.

### Transcription Factor Analysis

**FIGURE 5:**
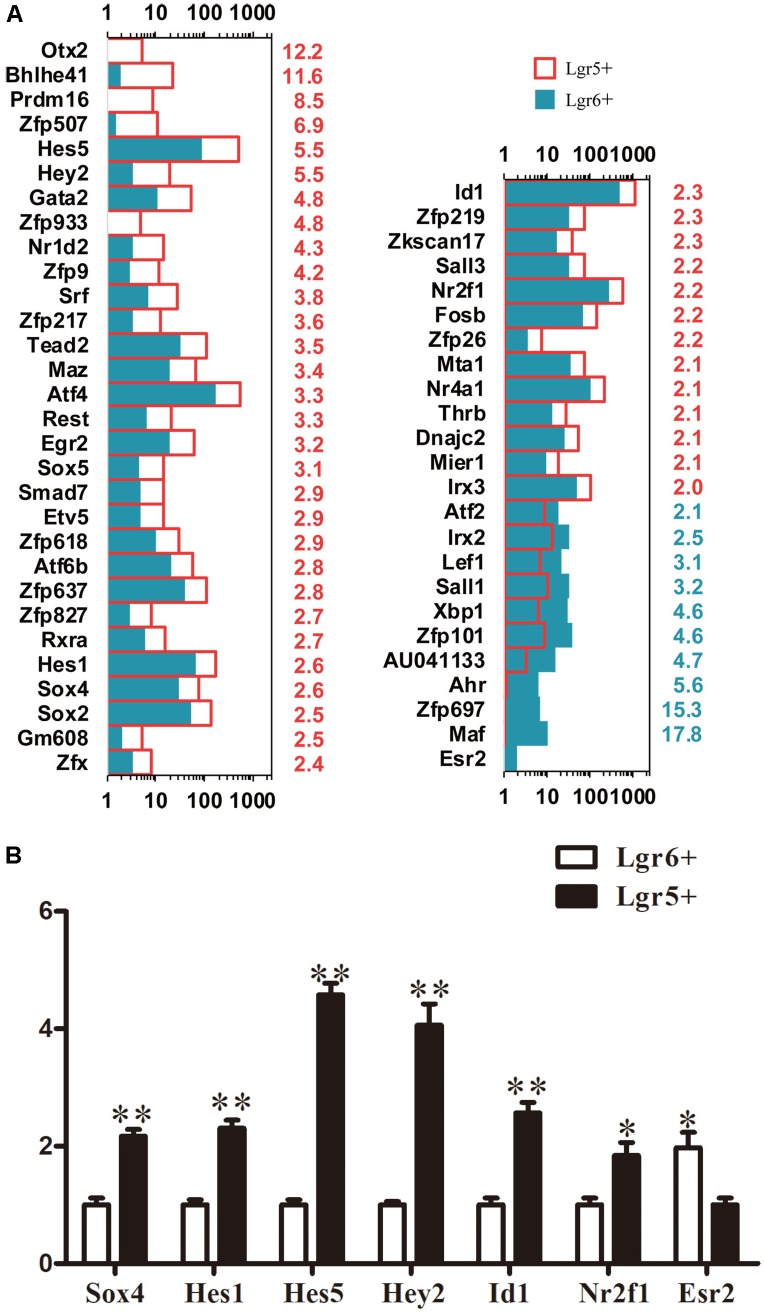
Genes regulating transcription factors. **(A)** Expression levels of differentially expressed TFs. The number in red on the right of each panel represents the fold difference in expression in Lgr5+ progenitors versus Lgr6+ progenitors, and the number in blue on the right of each panel represents the fold difference in expression in Lgr6+ progenitors versus Lgr5+ progenitors. **(B)** q-PCR analysis of the TF genes. ^∗^*p* < 0.05, ^∗∗^*p* < 0.01, *n* = 3.

Transcription factors (TFs) play vital roles during sensory epithelium development and the differentiation of progenitor cells into HCs. In order to determine which TFs might play roles in regulating the HC generation ability of these two HC progenitors, we examined 1,301 TF genes and found 54 significantly differentially expressed TF genes (*p* < 0.05). *Esr2* was significantly more highly expressed in Lgr6+ progenitors compared to the Lgr5+ progenitors (**Figure 5A**). *Hey2*, *Hes1*, *Hes5*, *Sox4*, *Id1*, and *Nr2f1* were significantly highly expressed in Lgr5+ progenitors. *Hey2* (Doetzlhofer et al., 2009), *Hes1*, and *Hes5* (Zine et al., 2001; Kelley, 2006) are transcriptional repressors that negatively regulate HC differentiation, and *Sox4* (Gnedeva and Hudspeth, 2015), *Id1* (Ozeki et al., 2007), and *Nr2f1* (Tang et al., 2006) play roles in the developing sensory tissues of the mammalian inner ear by regulating cell proliferation and HC formation. To verify the RNA-Seq data, qPCR was performed and the results were consistent with above RNA-Seq analysis (**Figure 5B**). The functions of many differentially expressed TFs in the proliferation and differentiation of these two HC progenitors are unclear and need to be researched in the future.

### Cell Cycle Analysis

**FIGURE 6:**
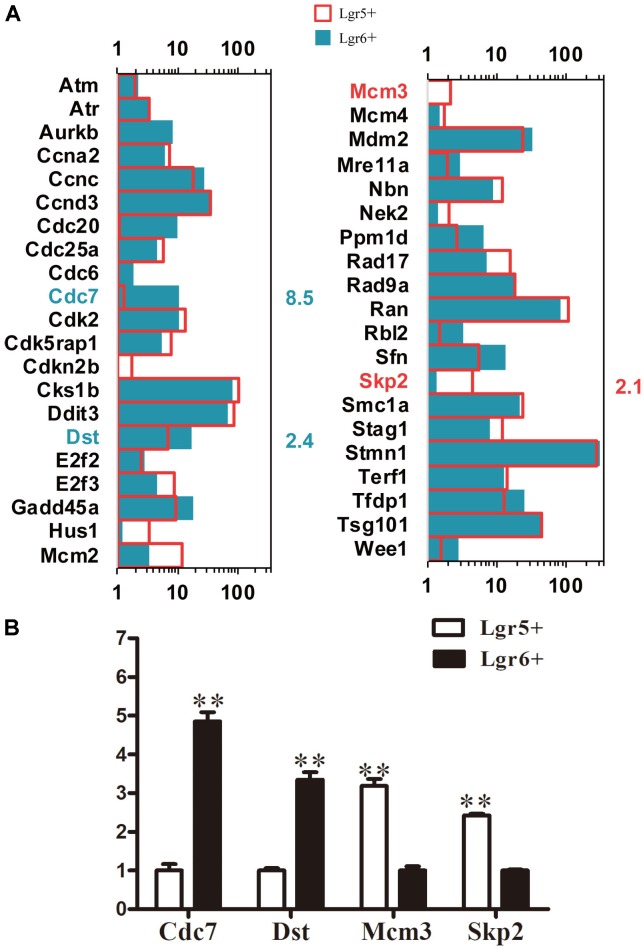
Cell cycle genes in Lgr5+ progenitors and Lgr6+ progenitors. **(A)** The expression of genes involved in the cell cycle in Lgr5+ progenitors and Lgr6+ progenitors. The significantly differentially expressed gene names in Lgr5+ progenitors and Lgr6+ progenitors are labeled in red and blue, respectively. The number in red on the right of each panel represents the fold difference in expression in Lgr5+ progenitors versus Lgr6+ progenitors, and the number in blue on the right of each panel represents the fold difference in expression in Lgr6+ progenitors versus Lgr5+ progenitors. **(B)** q-PCR analysis of the cell cycle genes. ^∗∗^*p* < 0.01, *n* = 3.

Cell cycle analysis was performed to explore the mechanism behind the difference of proliferation ability between these two HC progenitors. A total of 41 cell cycle genes were examined, and 4 significantly differentially expressed cell cycle genes were found (*p* < 0.05). *Dst* and *Cdc7* were highly expressed in Lgr6+ HC progenitors (**Figure 6A**), while *Mcm3* and *Skp2* were highly expressed in Lgr5+ HC progenitors (**Figure 6A**). The qPCR data were consistent with the above RNA-Seq results (**Figure 6B**).

### Signaling Pathway Analysis

**FIGURE 7:**
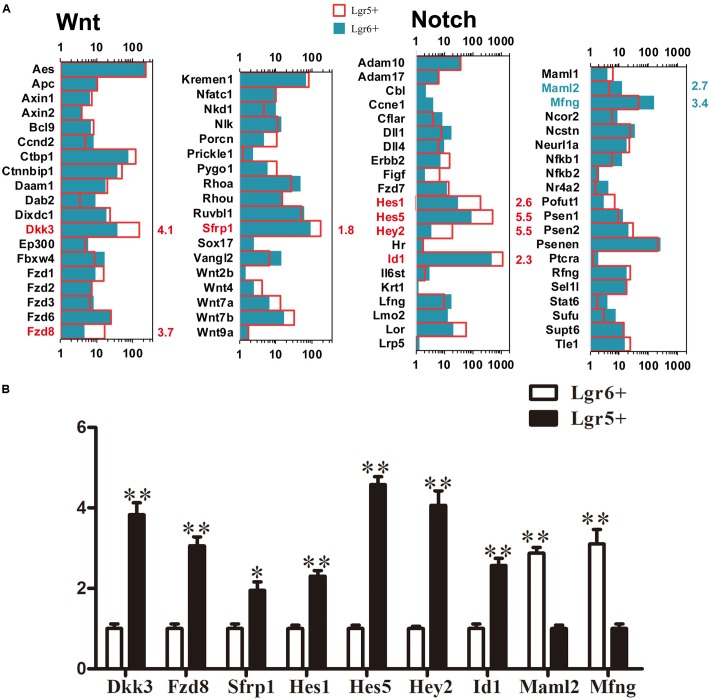
Signaling pathway genes in Lgr5+ progenitors and Lgr6+ progenitors. **(A)** The differentially expressed genes in Lgr5+ progenitors and Lgr6+ progenitors that are involved in the Wnt and Notch signaling pathways. The significantly differentially expressed genes in Lgr5+ progenitors and Lgr6+ progenitors are labeled in red and blue, respectively. The number in red on the right of each panel represents the fold difference in expression in Lgr5+ progenitors versus Lgr6+ progenitors, and the number in blue on the right of each panel represents the fold difference in expression in Lgr6+ progenitors versus Lgr5+ progenitors. **(B)** q-PCR analysis of the Notch and Wnt signaling pathway genes. ^∗∗^*p* < 0.01, *n* = 3.

*Lgr5* and *Lgr6* are both downstream target genes of the Wnt/β-catenin signaling pathway. We measured the expression levels of genes involved in the Notch and Wnt pathways and found 54 significantly differentially expressed genes between these two HC progenitors (*p* < 0.05, **Figure 7A**). The genes significantly increased in Lgr5+ progenitors compared to Lgr6+ progenitors included *Dkk3*, *Fzd8*, *Sfrp1*, *Hes1, Hes5*, *Hey2*, and *Id1*. *Hes1*, *Hes5*, *Hey2*, and *Id1* are target genes of Notch signaling, and this suggests that a lower level of Notch signaling in Lgr6+ progenitors might be responsible for their greater capacity for HC generation. The expression of the Notch signaling pathway genes *Maml2* and *Mfng* was significantly greater in Lgr6+ progenitors compared to Lgr5+ progenitors, but only *Mfng* has been reported in the development of the cochlea (Basch et al., 2016). Again, the q-PCR data were consistent with the above RNA-Seq analysis (**Figure 7B**).

## Discussion

*Lgr5* and *Lgr6*, which are encoded by Wnt target genes, are markers of adult stem/progenitor cells in many tissues. Lgr5 has been reported to mark stem cells in the small intestine, colon (Barker et al., 2007), stomach (Barker et al., 2010), hair follicle (Jaks et al., 2008), and cochlea (Chai et al., 2012); Meanwhile, in the skin (Snippert et al., 2010), nails (Lehoczky and Tabin, 2015), tongue (Ren et al., 2014), lung (Oeztuerk-Winder et al., 2012), and mammary gland (Blaas et al., 2016), Lgr6 is known to be a stem cell marker.

Our previous studies have reported the expression of Lgr5 (Chai et al., 2011) and Lgr6 (Zhang et al., 2015) in the developing inner ear. The expression of Lgr5 gradually decreases during the development and maturation of the cochlea, and the expression of Lgr5 in the IPs, the IPCs, and the lateral GER gradually disappears as the neonatal cochlea matures; in the adult cochlea, Lgr5 is expressed only in D3s. In the embryotic and neonatal mouse cochlea, Lgr6 is expressed in the IPs. With the maturation of the cochlea, Lgr6 expression is seen in both the IPs and the inner border cells (IBCs) (Zhang et al., 2015). No Lgr6 expression was observed in the mature cochlea (Zhang et al., 2015). Although the expression patterns of Lgr6 and Lgr5 share many differences, as well as some similarities, in the development of the cochlea, in general Lgr6 is expressed in a subpopulation of Lgr5+ progenitors in the embryonic and neonatal cochlea.

Multiple studies have demonstrated that Lgr5 marks the cochlear progenitors (Chai et al., 2012; Shi et al., 2013), and after HC damage Lgr5+ progenitors can generate new cochlear HCs via mitotic regeneration and/or direct differentiation in the neonatal stage (Chai et al., 2012; Shi et al., 2012, 2013; Jan et al., 2013; Cox et al., 2014). Our previous studies showed that Lgr6+ progenitors isolated from neonatal cochleae by flow cytometry are capable of generating HCs *in vitro*, which indicates that Lgr6+ cells are also cochlear HC progenitors (Zhang et al., 2015). However, a detailed comparison between Lgr6+ progenitors and Lgr5+ progenitors has not yet been undertaken. In this study, we found that isolated Lgr6+ progenitors showed a much greater capacity to generate HCs than Lgr5+ progenitors, while Lgr5+ progenitors showed greater capacity for proliferation and sphere-forming compared with Lgr6+ progenitors. In order to explore the detailed mechanisms leading to the differences of Lgr5+ and Lgr6+ progenitors, we analyzed the complete gene expression profiles of these two progenitor populations.

### Differentially Expressed Genes in Lgr5+ Progenitors and Lgr6+ Progenitors

Most of the top 100 differentially expressed genes between these two HC progenitors were not reported in the inner ear before. Five reported genes, including *Hes5*, *Hey2*, *Gata2*, *Fgfr2*, and *Stox1*, were highly expressed in Lgr5+ HC progenitors. *Hes5* and *Hes1* encode two inhibitory basic helix-loop-helix (bHLH) proteins that down-regulate the expression level of prosensory genes, including *Atoh1*, in differentiating cochlear SCs (Kelley, 2006). *Atoh1* is essential for the formation of HCs in the development of the cochlear epithelium (Bermingham et al., 1999), thus the high expression of *Hes5* in Lgr5+ progenitors might suppress the differentiation of progenitors into HCs. Similarly, *Hey2* has been reported to block Atoh1-induced differentiation of SCs into HCs (Doetzlhofer et al., 2009). *Fgfr2* plays a key role in mesenchymal-epithelial signaling during early organogenesis, and *Fgfr2*-null mice have developmental defects in the inner ear (De Moerlooze et al., 2000). *Gata2* is critical for vestibular organ morphogenesis (Haugas et al., 2010). *Stox1* is selectively expressed in the cochlear sensory epithelium, and overexpression of *Stox1* enhances cell proliferation and sphere formation (Nie et al., 2015), suggesting that *Stox1* might be one of the genes leading to the greater proliferation ability of Lgr5+ progenitors. Genes highly expressed in Lgr6+ progenitors compared to Lgr5+ progenitors include *Adk* and *Otoa*. Inhibition of Adk in the cochlea postpones the onset of age-related hearing loss (Vlajkovic et al., 2011). *Otoa* mutations are connected with autosomal recessive deafness type 22 (Zwaenepoel et al., 2002). Other than these, the rest of the top 100 differentially expressed genes have not been reported and their roles in the sensory epithelium need further research in the future.

### Transcription Factor Analysis

We examined 1,301 TF genes and found 54 significantly differentially expressed TF genes. Some reported TF genes, including *Hes1*, *Hes5*, *Hey2*, *Sox4*, *Id1*, and *Nr2f1*, are highly expressed in the Lgr5+ progenitors compared to Lgr6+ progenitors. *Sox4*, which is a member of the *SoxC* family of TF genes, has effects on the proliferation and differentiation process of the prosensory epithelium, and enhanced expression of *SoxC* genes restores proliferation in the adult utricular sensory epithelia (Gnedeva and Hudspeth, 2015). To confirm the qPCR verification of Sox4 expression, we also performed immunostaining experiments using the Sox4 antibody. We found that Sox4 was expressed in all of the SCs, including the first row of Deiters’ cells (DC1), the second row of Deiters’ cells (DC2), DC3, IPs, outer pillar cells(OPs), the lesser epithelial ridge (LER) and the GER in P3 cochlea, and there was no obvious expression difference between DCs and IPs in our immunostaining result (**Supplementary Figure S3**). However, immunostaining is not an accurate method to quantify the exact expression level of the protein. Id proteins are bHLH factors that inhibit cell differentiation and promote cell proliferation by negatively regulating many bHLH TFs, including Math1 (Benezra et al., 1990; Norton, 2000). *Id1* promotes the proliferation of OC1 cells and is involved in cochlear development (Ozeki et al., 2007). *Nr2f1* is expressed during cochlear development prior to and during the differentiation process of HCs and SCs (Tang et al., 2006). *Nr2f1* deficiency results in supernumerary HCs and SCs in the cochlear duct (Tang et al., 2006). In general, these reported TF genes that have higher expression in Lgr5+ progenitors are involved in cell proliferation and prosensory epithelial differentiation, and upregulation of these TF genes inhibits the differentiation of SCs into HCs. This might explain why the Lgr6+ progenitors have greater HC differentiation ability compared to Lgr5+ progenitors. Among the previously characterized TFs, *Esr2* is highly expressed in Lgr6+ progenitors compared to Lgr5+ progenitors. *Esr2* plays a role in preventing age-related hearing loss (Simonoska et al., 2009). However, the majority of the significantly differentially expressed TF genes need to be studied in detail in the inner ear.

### Cell Cycle Analysis

Two cell cycle-related genes, *Mcm3* and *Skp2,* are highly expressed in Lgr5+ progenitors. *Skp2* plays a critical role in cell proliferation and differentiation of the developing auditory system. *Skp2* positively regulates the G1-S transition through regulating p27 and thus triggers the proliferation of quiescent cochlear precursor cells (Dong et al., 2003; Minoda et al., 2007); however, during differentiation Skp2 expression is down-regulated in the auditory epithelia. This suggests that up-regulation of Skp2 might result in the significantly higher proliferation seen in Lgr5+ progenitors compared to Lgr6+ progenitors. In this study, we also found that some positive regulatory genes of the cell cycle (*Ccna2*, *Ccnd3*, *Ran*, *Stmn1*, and *Smc1a*) and negative regulatory genes of the cell cycle (*Cadkrap1* and *Mdm2*) are expressed in cochlear Lgr5+ progenitors and Lgr6+ progenitors. These newly discovered cell cycle regulatory genes need detailed characterization in the future.

### Signaling Pathway Analysis

Most of the genes for the Wnt and Notch signaling factors are expressed at similar levels in Lgr5+ progenitors and Lgr6+ progenitors. *Dkk3*, *Fzd8*, and *Sfrp1*, which are Wnt signaling pathway-related genes, have higher expression levels in Lgr5+ progenitors. *Dkk3* encodes a secreted protein that can interact with the Wnt signaling pathway and thus plays important roles in embryonic development processes (Ang et al., 2004; Diep et al., 2004). *Sfrp1* can directly interact with frizzled (Bafico et al., 1999) and activate Wnt/β-catenin signaling (Uren et al., 2000). *Fzd8*, a Wnt receptor that can activate both the canonical Wnt/β-catenin pathway and non-canonical Wnt signaling pathway, is reported to regulate the polarity and motility of neuroblasts (Kikuchi et al., 2007). However, the functions of *Dkk3*, *Fzd8*, and *Sfrp1* in the cochlea require further studies in greater detail. *Hes5*, *Hes1*, *Hey2*, and *Id1*, which are Notch signaling-related genes, have higher expression levels in Lgr5+ progenitors. The functions of these genes have been discussed above. The increased expression of *Hes1*, *Hes5*, and *Hey2* in Lgr5+ progenitors might suppress the differentiation of Lgr5+ progenitors into HCs (Doetzlhofer et al., 2009). *Id1* was shown to positively regulate the proliferation of cochlear sensory epithelial cells (Ozeki et al., 2007), and the upregulation of *Id1* in Lgr5+ progenitors might promote the proliferation of Lgr5+ progenitors. The Notch signaling pathway-related genes that have higher expression levels in Lgr6+ progenitors include *Mam2* and *Mfng*. *Mfng*, which is one of the Notch modifiers, is transiently expressed specifically in the future inner HC region and is required to regulate the differentiation of inner HCs and IPCs (Basch et al., 2016), but the functions of *Mam2* in the inner ear need to be studied further. Activating Wnt signaling can enhanced SC proliferation (Chai et al., 2012; Shi et al., 2014), while inhibiting the Notch pathway can promote HC differentiation and activating the Notch pathway can lead to the overproduction of SCs (Kiernan, 2013). Our results suggest that it might be the combined functions of Wnt and Notch signaling that lead to the increased proliferation capacity of Lgr5+ progenitors and the increased differentiation capacity of Lgr6+ progenitors (Murata et al., 2012; Munnamalai and Fekete, 2013).

## Conclusion

Our results show that compared to the Lgr5+ progenitors, Lgr6+ progenitors have significantly greater capacity to generate HCs and thus are an enriched HC progenitor population, while Lgr5+ progenitors have greater capacity to proliferate than Lgr6+ progenitors. Furthermore, our RNA-Seq data provide a handful of genes that might play roles in the proliferation and differentiation of cochlear progenitors, and these data will be very useful in designing strategies for promoting hair cell regeneration and hearing loss therapy.

## Data Accession

The raw data are available on NCBI GEO database, accession number GSE114034.

## Author Contributions

YZ, HL, and RC designed the study. YZ, LG, and XL performed the laboratory experiments. YZ, HL, RC, LG, XL, YC, SZ, MT, SS, and LZ contributed to critical discussion and data analysis. YZ, HL, LG, YC, and RC wrote the paper. All authors read and approved the final manuscript.

## Conflict of Interest Statement

The authors declare that the research was conducted in the absence of any commercial or financial relationships that could be construed as a potential conflict of interest.

## Supplementary Material

FIGURE S1 Expression levels of the top 200 genes in Lgr5+ progenitors and Lgr6+ progenitors. **(A)** Expression levels of the top 200 genes in Lgr5+ progenitors in descending order. Numbers in red on the right side of each panel represent the ranking of the same genes in Lgr6+ progenitors. **(B)** Expression levels of the top 200 genes in Lgr6+ progenitors in descending order. Numbers in blue on the right side of each panel represent the ranking of the same genes in Lgr5+ progenitors.

FIGURE S2 Lgr5 and Lgr6 expression in the sensory epithelium of P3 cochleae. At P3, there was only one row of Lgr6+ cells in the middle and basal turns of the cochlea, but Lgr6+ did not expressed in the apical turn of cochlea, while Lgr5 expression was similar from the apex to the base in the sensory epithelium.

FIGURE S3 Sox4 expression in the sensory epithelium at P3 cochlea. At P3, Sox4 expressed in all of SCs, including first row of Deiters’ cells (DC1), the second row of Deiters’ cells (DC2), DC3, IP, outer pillar cells(OP), the lesser epithelial ridge (LER) and the GER.

TABLE S1 The Primers for the q-PCR assay.
