# Supplementary figures and images for: Characterization of Lgr6+ Cells as an Enriched Population of Hair Cell Progenitors Compared to Lgr5+ Cells for Hair Cell Generation in the Neonatal Mouse Cochlea
Source: Front Mol Neurosci. 2018 May 14;11:147. doi: 10.3389/fnmol.2018.00147 (PMC5961437; doi:10.3389/fnmol.2018.00147)

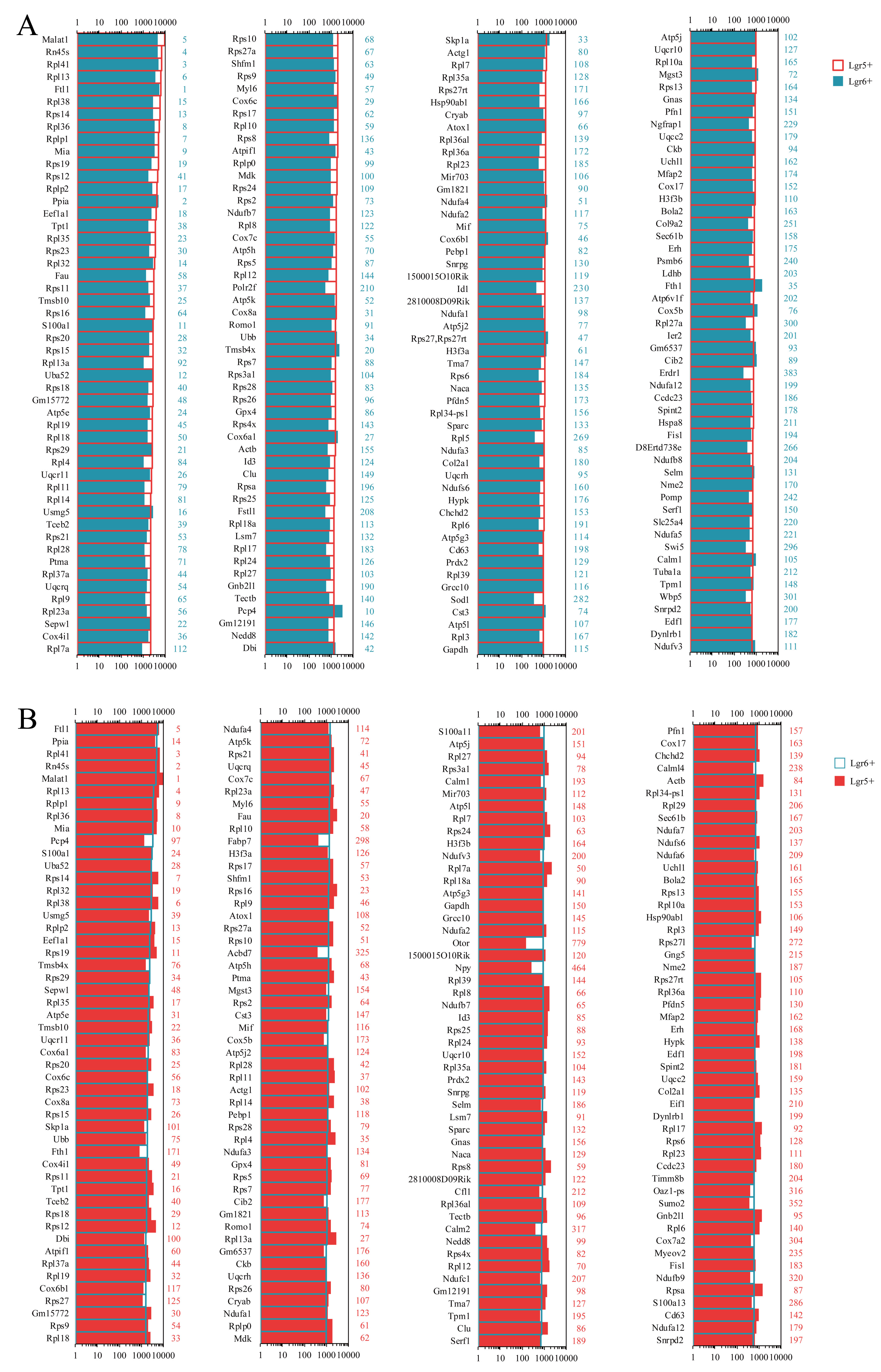

Supplement: FIGURE S1 — Expression levels of the top 200 genes in Lgr5+ progenitors and Lgr6+ progenitors. (A) Expression levels of the top 200 genes in Lgr5+ progenitors in descending order. Numbers in red on the right side of each panel represent the ranking of the same genes in Lgr6+ progenitors. (B) Expression levels of the top 200 genes in Lgr6+ progenitors in descending order. Numbers in blue on the right side of each panel represent the ranking of the same genes in Lgr5+ progenitors. [file Image_1.jpeg]

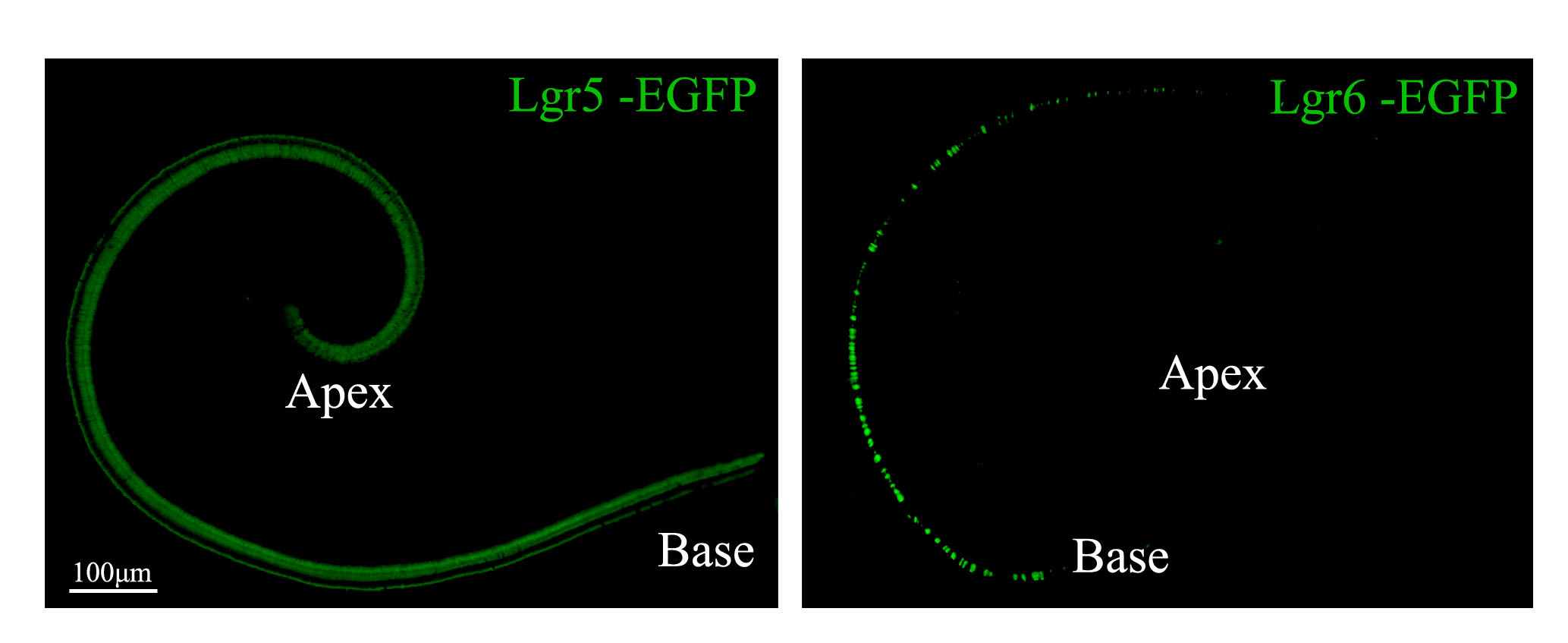

Supplement: FIGURE S2 — Lgr5 and Lgr6 expression in the sensory epithelium of P3 cochleae. At P3, there was only one row of Lgr6+ cells in the middle and basal turns of the cochlea, but Lgr6+ did not expressed in the apical turn of cochlea, while Lgr5 expression was similar from the apex to the base in the sensory epithelium. [file Image_2.jpeg]

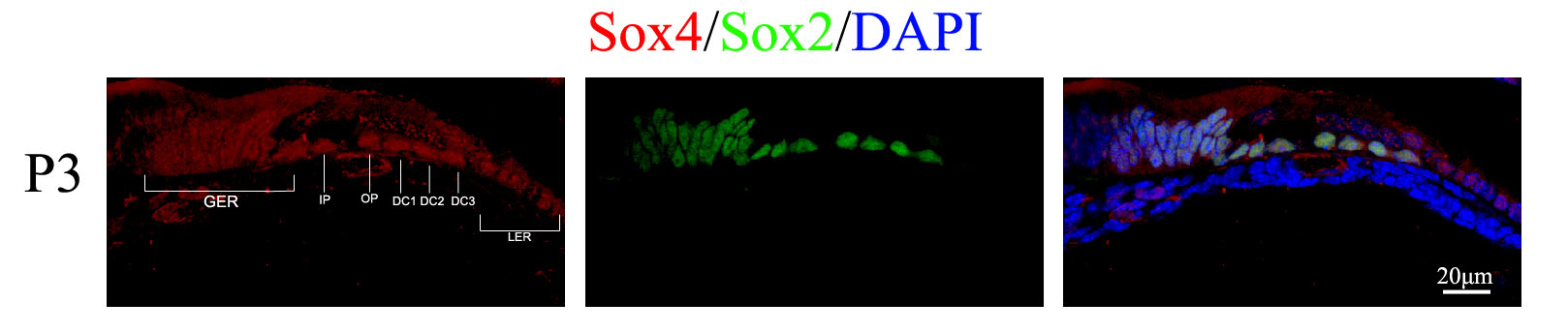

Supplement: FIGURE S3 — Sox4 expression in the sensory epithelium at P3 cochlea. At P3, Sox4 expressed in all of SCs, including first row of Deiters’ cells (DC1), the second row of Deiters’ cells (DC2), DC3, IP, outer pillar cells(OP), the lesser epithelial ridge (LER) and the GER. [file Image_3.jpeg]
