# Supplementary material for: Characterization of Lgr6+ Cells as an Enriched Population of Hair Cell Progenitors Compared to Lgr5+ Cells for Hair Cell Generation in the Neonatal Mouse Cochlea
Source: Front Mol Neurosci. 2018 May 14;11:147. doi: 10.3389/fnmol.2018.00147 (PMC5961437; doi:10.3389/fnmol.2018.00147)
Supplement: TABLE S1 — The Primers for the q-PCR assay. [file Table_1.pdf]

Gene	Forward sequence	Reverse sequence
Lgr6	GTA TGA ACA ACC TCA CGG AGC	TTG GAG GCC AGA GAA TGC C
Sox2	ATG AAC GGC TGG AGC AAC GGC A	TCA CAT GTG CGA CAG GG GCA GT
Lgr5	TCT TCA CCT CCT ACC TGG ACC T	GGC GTA GTC TGC TAT GTG GTG T
Brn3.1	CCC AAA TTC TCC AGC CTA CAC	GGC GAG ATG TGC TCA AGT AAG T
Hes1	ACG ACA CCG GAC AAA CCA	ATG CCG GGA GCT ATC TTT CT
Hes5	TGC TCA GTC CCA AGG AGA AA	AGC TTG GAG TTG GGC TGG T
Nr2f1	AAT ACT GCC GCC TCA AGA AG	CGC AGC AGC AGA GAA ATG TA
Skp2	GCT GCC CTC GGT CCT TTA T	TTG G AGC ACT CGG ACA GAA T
Id1	CCT GCT CTA CGA CAT G AA CG	TTC AGA CTC CGA GTT CAG CT
Dkk3	CAA AGG TGG CAA TGG GAC C	CAG TTC CCA GGT GAT GAG A
Cdc7	TGC T TG CCG TAT CTG GAA CA	CCA AAC CGA AGT CCA CCA AG
Mfng	ACC GTG ATG TCT ATG TGG GC	TGA GAG CAG AAG TGT CCA CA
Dst	CCA CGT GGT CAC TAT GTC CG	CGA TGA TGA GTT T GC CCC AC
Sox4	AC ACC CTG CCG ACA AGA AA	TCC ATC TTC GTA CAA CCC CA
Hey2	GAT CAT TTG AAG ATG CTC CAG G	TCA AGC ACT CTC GGA ATC CA
Fzd8	CTG GCA TGA AGT GGG GTA ATG	CGT AGG TTG TCA AGG CTC TG
Esr2	TTG TGC CAG CCC TGT TAC TA	ACT GAT TCG TGG CTG GAC A
Mcm3	TGG AGC GGG AAC TTG AAA AC	CAA AGC ACG TAC CGC AGA AG
Maml2	GGT CAC CCT TAC AAC TTC AGC	GGA CTG CTG GGA GTT CAT GT
Sfrp1	AC CAC GGA AGC CTC TAA GC	AGG GTT TCT TCT TCT TGG GGA
Gapdh	AAC GGG AAG CCC ATC ACC ATC TT	CAG CCT TGG CAG CAC CAG TGG
